# What Can Physiology Tell Us about State of Interest?

**DOI:** 10.3390/jintelligence12080079

**Published:** 2024-08-16

**Authors:** Ksenia Babanova, Victor Anisimov, Alexander Latanov

**Affiliations:** 1 Faculty of Biology, Lomonosov Moscow State University, 119992 Moscow, Russia; shedenko.ksyu@gmail.com (K.B.); latanov@neurobiology.ru (A.L.); 2 Research Institute of Functional Brain Development and Peak Performance, Peoples’ Friendship University of Russia, Miklukho-Maklaya str.6, 117198 Moscow, Russia

**Keywords:** eye movements, eye tracking, gaze direction, attention, reading, interest

## Abstract

The state of interest as a positive emotion is associated with the ability to comprehend new information and/or to better consolidate already perceived information, to increase the attention level to the object, to increase informational processing, and also to influence such processes as learning and motivation. The aim of this study was to reveal oculomotor correlates that can predict the locus of interest in cases of people perceiving educational information from different areas of knowledge presented as text or multimedia content. Sixty (60) volunteers participated in the study (50% males, mean age 22.20 ± 0.51). The stimuli consisted of 16 texts covering a wide range of topics, each accompanied by a comprehension question and an interest assessment questionnaire. It was found that the multimedia content type triggered more visual attention and gave an advantage in the early stages of information processing. The first fixation duration metric for the multimedia stimuli allowed u to characterize the subjective interest assessment. Overall, the results suggest the potential role of eye-tracking in evaluating educational content and it emphasizes the importance of developing solutions based on this method to enhance the effectiveness of the educational process.

## 1. Introduction

The main role of emotions, due to their high information specificity compared to undifferentiated arousal, is their ability to activate adaptive behavior that could influence human survival (Izard 2009). In this regard, the identification and regulation of emotions and emotional disorders are necessary for maintaining mental health and normal functioning in society (Hu et al. 2017; Zhu et al. 2019). Positive emotions broaden an individual’s momentary thought–action repertoire (Fredrickson 2004). Despite the importance of positive emotions for behavior, their physiological mechanisms (Hu et al. 2017; Behnke et al. 2022), as well as several aspects of their manifestation at the physiological level, remain not fully understood.

The positive emotion of interest is experienced by individuals more frequently than other emotions, eliciting a desire to explore (Fredrickson 2004; Izard 2009) in order to obtain new information and/or a better understanding of existing information (Reeve et al. 2015). Interest is associated with increased attention to stimuli and it plays a crucial motivational role in the formation and development of skills, abilities, and intellect (Izard 2009; Reeve et al. 2015; Renninger and Hidi 2019). Practical observations show that interest is linked to increased productivity (Izard 2009; Reeve et al. 2015; Diener et al. 2020) and academic performance (Jansen et al. 2016; Fryer and Ainley 2019). Interest is essential for creativity (Izard 2009), and it is associated with self-confidence, competence (Fryer and Ainley 2019), and job satisfaction (Diener et al. 2020); it can be considered a biological force that influences the capacity for learning (Renninger and Hidi 2022).

Unlike other motivational states, interest has a pronounced specificity towards content (Krapp 2002; Hidi and Renninger 2006). The “object of interest” can be a specific subject, theme, abstract idea, or any other type of content that has a cognitive representation in the psychological relationship between the individual and the environment (Krapp 2002). Within the framework of experimental studies on reading-induced interest, the term “topic of interest” is used to describe a long-term predisposition to interact with specific thematic content (Krapp 2002). Motivation for reading depends not only on its positive “intrinsic value” but also on its “negative value” (Eccles and Wigfield 2020).

A wide range of methods for measuring interest is presented in the contemporary scientific literature (Rowland et al. 2019). Existing interest state assessment questionnaires contain seven to 26 questions (Rotgans 2015; Luo et al. 2019; Knekta et al. 2020; Wang et al. 2021; Dahl and Nierenberg 2021; Kleespies et al. 2021; Potvin et al. 2022). The questions are based on evaluating various factors: significance (Luo et al. 2019; Knekta et al. 2020; Dahl and Nierenberg 2021; Kleespies et al. 2021; Potvin et al. 2022), positive emotions (Luo et al. 2019; Knekta et al. 2020; Dahl and Nierenberg 2021; Kleespies et al. 2021; Potvin et al. 2022), knowledge (Luo et al. 2019; Kleespies et al. 2021), engagement (Luo et al. 2019; Potvin et al. 2022), interaction repeatability (Knekta et al. 2020), overall interest, situational dependency, competency, desire for competence development, self-regulation (Dahl and Nierenberg 2021), pleasure, attention, and usefulness (Potvin et al. 2022).

However, such questionnaires cannot be applied in a real-time or pseudo real-time mode to provide information on the specificity of the content that does or does not attract interest. This information may be crucial for timely material adjustment to meet students’ needs and for the development of corresponding technical solutions.

Eye-tracking is a non-invasive technique that estimates the distribution of visual attention. Objectively recorded eye movement indicators can be potentially used to determine interest in the context of learning; specifically, in the most popular versions of educational content—text and illustrated text. The combined presentation of multiple objects in the visual field, including text and images, leads to a change in the distribution of visual attention towards each object (Johnson and Mayer 2012; Mayer et al. 2014; Mason et al. 2017; Lindner et al. 2021), which in the field of education is referred to as the “multimedia effect”. The multimedia effect is associated with improved testing outcomes and increased interest among learners.

Therefore, in this article, we assume that interest and related to interest subjective measures can be detected using a non-invasive technique—eye-tracking. However, visual attention will differ for purely textual and multimedia content.

## 2. Materials and Methods

### 2.1. Participants

The volunteers that participated in this study were students and staff of higher educational institutions in Moscow aged 18 to 33 years. A total of 60 participants took part in the study; all were right-handed and the left eye was dominant in 50% of cases. Exclusion criteria included the intake of antibiotics or psychotropic drugs, epileptic status, and head injuries. The experiment was conducted in accordance with the Helsinki Declaration, and ethical approval was obtained from the MSU bioethical committee. Each volunteer was familiarized with the research procedure and signed an informed consent to participate in the experiment.

### 2.2. Stimuli

Sixteen educational texts about biology, chemistry, programming, engineering, psychology, journalism, economics, philosophy, music, visual arts, architecture, data science and management, and education were used. The texts did not have a strongly negative connotation and did not contain socially or ethically unacceptable information. The content of the original texts was edited to present a complete thought within the specified volume.

The size of the model texts was 1866.81 ± 5.44 characters and 242.81 ± 5.04 words. The readability of the texts was calculated according to existing complexity indices adapted for the Russian language. The Automated Readability Index (ARI) was 15.70 ± 1.14 points, the Simple Measure of Gobbledygook (SMOG) index was 13.68 ± 1.00 points, and the Flesch Readability Index was 13.68 ± 1.00. The texts were aligned to the left to maintain an equal distance between letters and words.

**Figure 1:**
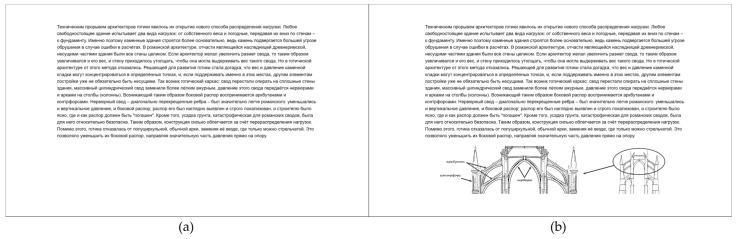
An example of experimental stimuli: (**a**) TEXT; (**b**) TEXT + PICTURE.

Each text was accompanied by an image illustrating the main idea of the text. Original images from sources were redrawn in a uniform style in black and white. The materials were presented in two different formats (Figure 1): in the first case (TEXT), the text was not accompanied by an illustration, leaving the area under the image blank, and in the other case, an image was added to the text (TEXT + PICTURE).

The material was presented on the screen of a demonstration monitor with a width of 527 mm, a height of 298 mm, and a diagonal of 23 inches. The distance to the respondent’s eyes of no less than 60 cm was chosen based on the equipment manufacturer’s recommendation. The horizontal angular coverage of the text area was 34.88 degrees, and vertically it was 30.93 degrees. The image was positioned below the text, occupying an area with the same horizontal angular coverage as the text. The vertical angular coverage of the image was 16.13 degrees. The text was printed in a non-serif Arial Regular 18 font size, with a lettering height of 1.24 degrees and an average width of one character horizontally at 0.31 degrees.

For each text, one question reflecting the main idea of the text was formulated. All four answer choices included detailed explanations of the main idea of the text; however, the distractors contained factual errors.

### 2.3. Interest Assessment

Based on a comparison with existing questionnaires (Rotgans 2015; Luo et al. 2019; Knekta et al. 2020; Dahl and Nierenberg 2021; Kleespies et al. 2021; Potvin et al. 2022) aimed at determining the level of interest, the 7 most common statements were selected and translated to Russian. The reverse translation of the expressions used was as follows:

- “I am familiar with the topic of this text” (“familiarity”, Q1);
- “The information in this text was new to me” (“novelty”, Q2);
- “I found it easy to read this text” (“complexity”, Q3, inverted scale);
- “I want to learn more information on this topic” (“cognitive”, Q4);
- “I consider this topic personally important” (“value”, Q5);
- “I experience positive emotions when reading texts on this topic” (“emotions”, Q6);
- “I found it interesting to read this text” (“interest”, Q7).

Responses to each question were made on a seven-point Likert scale from “completely disagree” to “completely agree” (scored 1–7, respectively). Answers were duplicated in the form of emojis and text to allow respondents to more effectively compare their subjective assessment with the answer options.

### 2.4. Design and Procedure

After receiving information about the research procedure and obtaining informed consent, the determination of the profile of interhemispheric asymmetry was conducted. The determination of the dominant hand was performed using a shortened version of the Annett questionnaire (Annett 1970), while the dominant eye was determined using the Dolman method (Cheng et al. 2004).

Subsequently, sensor installation and briefing took place. The key instruction was as follows: “*Texts will be presented on the screen to read at your comfortable pace. Read each text once, and after finishing reading, press the right side of the screen to proceed to the questions. Questions will appear after each text, and responses are required*”.

Experiment was balanced by gender and stimuli type (Figure 1). Stimuli were presented in a pseudorandom order. Questions were presented immediately after text reading. The experiment duration was different due to different reading speeds of participants (40–60 min).

### 2.5. Experiment Setup

The experimental setup included a location for the participant and separated location for the experimenter. The participant’s location consisted of an equipped room with soundproofing, a monitor for presenting stimuli, and a computer mouse. Eye movement recording was conducted using Neurobarometer 2.2.12 software–hardware complex (manufacturer—Neurotrend JSC, produced by Medikom-MTD LLC, Taganrog, Russia).

The N-TREND EyeTracker was used to record eye movements, with a sampling frequency of 500 Hz. The accuracy of gaze direction determination was 0.4 degrees, and it featured infrared illumination. The eye tracker was positioned beneath the monitor without additional means of respondent position fixation, as signal correction is provided during recording when the respondent’s eyes move within the boundaries of ±25 cm horizontally and ±13 cm vertically relative to the central position. The minimum fixation duration was 40 ms, the minimum distance between fixations was 0.8 degrees, and the minimum blink duration was 75 ms. At the beginning of the study, eye-tracker calibration was conducted. Nine calibration stimuli were positioned on the screen at nodes of an invisible grid. Calibration procedure was repeated until reaching the maximum quality in terms of the formal criterion of accuracy and data registration precision.

### 2.6. Eye Movements Analysis

Two main zones were identified for eye movement analysis: text and image. Fixations occurring in other areas were not taken into account in the analysis. Additionally, a fixation duration filter was implemented within the range of 80–800 ms.

Text and picture areas total fixation duration (average duration of fixations), text and picture areas total viewing time (sum of all fixations in the area of interest), total stimulus viewing time, and the number of transitions between areas (for TEXT + PICTURE stimuli) were calculated. For the text area, the average duration of the first fixation was also calculated. This metric represents the average duration of the first fixation on a word if it is a single fixation or the duration of the first fixation on a word if there are two or more consecutive fixations on that word.

## 3. Results

### 3.1. Subjective Assessment and Interest

A correlational analysis was conducted to understand the relationship between the answers. The results are presented in Table 1. Responses to all questions are significantly correlated with each other with varying strengths. For the subjective assessment of interest, moderate and strong correlations were found with the subjective assessment of complexity (rho = −0.69, *p* < 0.01), the desire to learn more about the topic (rho = 0.74, *p* < 0.01), the evaluation of the topic’s significance and importance (rho = 0.56, *p* < 0.01), and the evaluation (0.77, *p* < 0.01).

**Figure 2:**
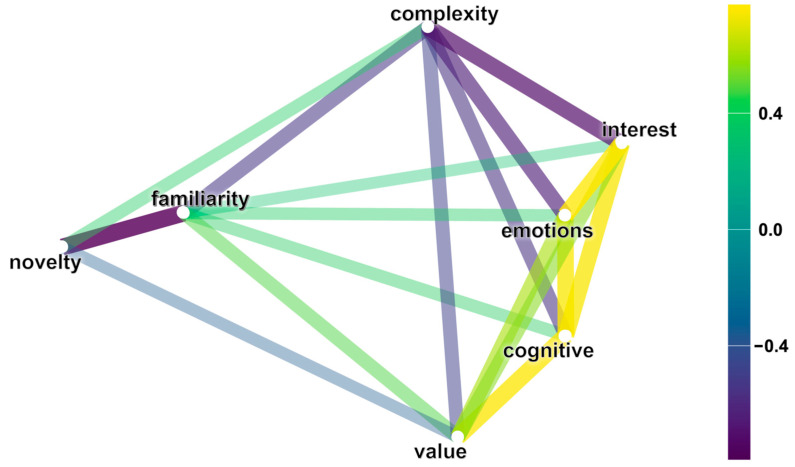
Graphical representation of the correlations between subjective assessment scales.

Based on the conducted correlation analysis, a graphical representation of the relationships depending on the sign and strength of the correlation was created (Figure 2). The closest connection to interest is observed with cognitive, emotional, and complexity components. Complexity, value, and familiarity are linked to all other subjective assessment scales. Novelty linked to complexity, familiarity, and value.

### 3.2. Multimedia Effect

#### 3.2.1. Subjective Assessment

**Figure 3:**
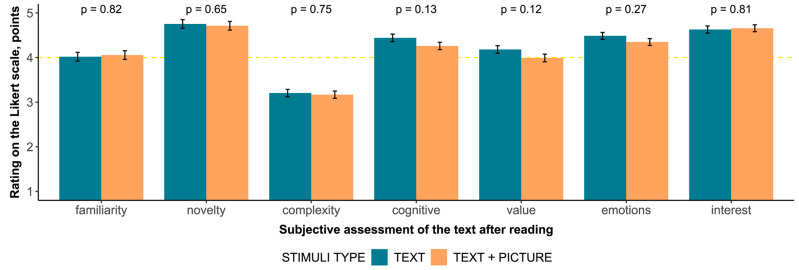
A different test of categories and Likert scale score for each of them.

To describe the differences in the perception of TEXT and TEXT + PICTURE, a comparison of scores for all the categories of the subjective assessment was conducted (Figure 3). No significant differences in the ratings were found. The distribution for each scale, regardless of type, differs significantly from random (all χ^2^ > 73.256, df = 6, *p* < 0.001).

The frequency of responses regarding the comparison of the two types of materials in terms of interest (χ^2^ = 3.782, df = 6, *p* = 0.706), cognitive (χ^2^ = 3.733, df = 6, *p* = 0.713) and emotional (χ^2^ = 4.456, df = 6, *p* = 0.615) components, perceived importance (χ^2^ = 5.137, df = 6, *p* = 0.526), difficulty (χ^2^ = 6.213, df = 6, *p* = 0.400), and familiarity with the topic (χ^2^ = 7.168, df = 6, *p* = 0.306) do not differ significantly.

However, the frequency distribution of responses to the question about the novelty of information differs at a trend level (χ^2^ = 11.306, df = 6, *p* = 0.080). A more detailed analysis found that the stimuli of type TEXT + PICTURE are less frequently judged as new (χ^2^ = 8.276, df = 3, *p* = 0.041).

#### 3.2.2. Eye-Tracking Metrics

The eye movement metrics were examined across stimuli types (Table 2). It was found that the duration of the first fixation on the text area, fixation duration on the text area, and text area total time do not differ significantly.

TEXT + PICTURE stimuli are associated with more visual attention than TEXT stimuli in the meaning of the total viewing time. The text reading time demonstrates no significant difference across the types.

The TEXT + PICTURE stimuli fixation duration on the image area was longer than on the text area (267 (51) ms compared to 240 (29) ms, F(1, 56) = 9.06, *p* < 0.001, η_2_g = 0.18). The total viewing time of the image was 5454 (3986) ms. The average number of switches between the text and image areas was 2.93 (5.89).

#### 3.2.3. Comprehension

The distribution of correct answers differs from random (514 correct, 337 incorrect, χ^2^ = 36.814, df = 1, *p* < 0.001). No significant difference was found in the frequency of correct answers depending on the stimuli type (χ^2^ < 0.001, df = 1, *p* = 1).

The proportion of correct answers regardless of the material type is associated with all subjective assessments, except for emotions (χ^2^ = 10.690, df = 6, *p* = 0.098); namely, familiarity (χ^2^ = 22.573, df = 6, *p* < 0.001), novelty (χ^2^ = 13.079, df = 6, *p* = 0.042), complexity (χ^2^ = 22.957, df = 6, *p* < 0.001), cognitive (χ^2^ = 16.780, df = 6, *p* = 0.010), value (χ^2^ = 24.940, df = 6, *p* < 0.001), and interest assessment (χ^2^ = 17.514, df = 6, *p* = 0.008).

**Figure 4:**
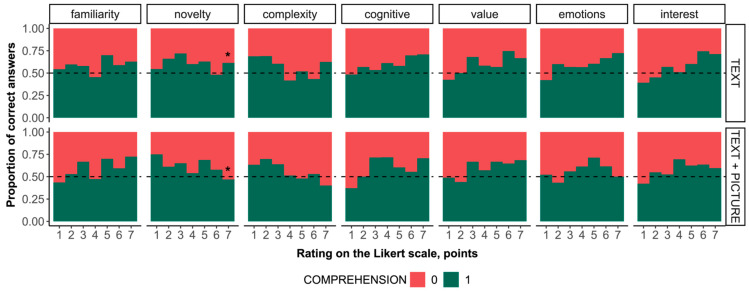
Distribution of the proportion of correct answers for TEXT and TEXT + PICTURE depending on the scores in each category of subjective assessment. * indicates *p* = 0.049.

The proportion of correct answers by subjective assessment and stimuli types is shown in Figure 4. For both stimuli types, the distribution of correct answers is associated with the subjective assessment of complexity (TEXT + PICTURE: χ^2^ = 13.485, df = 6, *p* = 0.036; TEXT: χ^2^ = 13.877, df = 6, *p* = 0.031) and value (TEXT + PICTURE: χ^2^ = 13.635, df = 6, *p* = 0.034; TEXT: χ^2^ = 15.410, df = 6, *p* = 0.017).

For the TEXT + PICTURE stimuli, but not for the TEXT stimuli, the distribution of correct answers is associated with the subjective assessment of familiarity (χ^2^ = 19.396, df = 6, *p* = 0.004), novelty (χ^2^ = 14.644, df = 6, *p* = 0.0232), cognitive (χ^2^ = 18.263, df = 6, *p* = 0.006), and emotions (χ^2^ = 13.538, df = 6, *p* = 0.035).

For the TEXT stimuli, but not for the TEXT + PICTURE stimuli, the distribution of correct answers is associated with the subjective interest assessment (χ^2^ = 18.877, df = 6, *p* = 0.004).

To assess the strength and direction of the relationships between the proportion of correct answers and subjective assessment, a correlation analysis was conducted (Table 3). The comparison of the results from two statistical methods indicates not only a difference in the relationship between the proportion of correct answers and subjective assessments, but also a distinct nature of the relationship for the two types of stimuli (linear or nonlinear).

Specifically, the relationship between the proportion of correct answers and the subjective assessment of complexity for the type TEXT is nonlinear, whereas for type TEXT + PICTURE, it is linear. Moreover, a strong positive linear relationship between the subjective assessment of interest and the number of correct answers is observed only for the TEXT type stimuli, but not for the TEXT + PICTURE type.

### 3.3. Visual Attention Distribution

Total viewing time is an integral behavioral characteristic of the interaction of a person with information. The TEXT stimuli total viewing time (text area total time) reflects changes in the subjective assessment of the familiarity (H(6, 403) = 39.065, *p* < 0.001), novelty (H(6, 403) = 25.407, *p* < 0.001), cognitive (H(6, 403) = 13.956, *p* = 0.030), and value (H(6, 403) = 16.345, *p* = 0.012) assessments, but not the difficulty, emotions, and interest assessments.

For the TEXT + PICTURE stimuli, both the total viewing time and text area total time reflect the subjective assessments of familiarity (H(6, 406) = 27.910, *p* < 0.001 and H(6, 406) = 28.508, *p* < 0.001, respectively) and novelty (H(6, 406) = 17.706, *p* = 0.007 and H(6, 406) = 17.954, *p* = 0.006, respectively). For other subjective measures, differences were not found (all *p* > 0.176).

**Figure 5:**
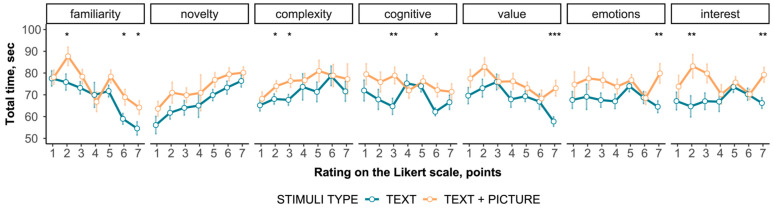
The dependence of the total viewing time on the subjective assessment of content. * indicates *p* < .05. ** indicates *p* < .01. *** indicates *p* < .001.

The relationship between the total viewing time and the subjective assessment is shown in Figure 5. Total time shows an almost monotonic increasing trend for the novelty metric, except for the neutral score. However, for all other metrics, total time showed mixed results. No correlations (rho > 0.3) were found for both the total viewing time and text area total time (Appendix A).

The viewing time of the image area is not associated with any of the scales of subjective assessment (all *p* > 0.195). The number of transitions between the text and image areas is linked to the perceived value (H(6, 343) = 15.989, *p* = 0.014) and it shows a trend-level association with the subjective assessment of the cognitive component (H(6, 343) = 11.378, *p* = 0.077). No significant differences were found for the other scales of subjective assessment (all *p* > 0.114). Thus, unlike the TEXT type, for the TEXT + PICTURE, the evaluation of the cognitive and valuable components is more likely related to the integration of information from the text and image areas than to the text itself.

### 3.4. Eye Movements Events

**Figure 6:**
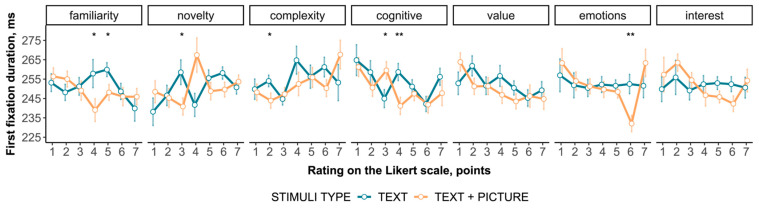
The dependence of the duration of the first fixation on the subjective assessment of content. * indicates *p* < .05. ** indicates *p* < .01.

Eye movement metrics (text and picture total fixation duration and first fixation duration for the text area) were analyzed in relation to their association with the subjective assessment for the two types of stimuli. The duration of the first fixation reflects information processing during reading (Figure 6).

For the TEXT stimuli, both the total fixation duration on the text area and the first fixation duration significantly change with different assessments of familiarity (H(6, 403) = 20.988, *p* = 0.002 and H(6, 333) = 12.658, *p* = 0.049, respectively) and the novelty (H(6, 403) = 21.992, *p* = 0.001 and H(6, 333) = 14.620, *p* = 0.023, respectively). The first fixation duration reflects the complexity assessment (H(6, 333) = 14.558, *p* = 0.024) while there is only a tendency for the total fixation duration on the text area (H(6, 403) = 10.791, *p* = 0.095). The first fixation duration, but not total fixation duration, reflects dependencies on the cognitive assessment (H(6, 333) = 15.462, *p* = 0.017). Both eye movement metrics for the TEXT type do not show a link to the value, emotions, and interest assessments.

For the TEXT + PICTURE stimuli, unlike the TEXT type, both the total fixation duration on the text area and the first fixation duration significantly do not show any dependencies on the familiarity and novelty assessments. The absence of a dependency or just a tendency is shown for the two metrics regarding the assessment of complexity (H(6, 406) = 7.500, *p* = 0.277 and H(6, 343) = 11.399, *p* = 0.077, respectively) and value (H(6, 406) = 8.989, *p* = 0.174 and H(6, 343) = 11.588, *p* = 0.072). Both metrics show a link to the cognitive (H(6, 406) = 17.287, *p* = 0.008 and H(6, 343) = 15.606, *p* = 0.016, respectively) and emotional (H(6, 406) = 18.195, *p* = 0.006 and H(6, 343) = 23.692, *p* < 0.001, respectively) assessments. The first fixation duration, but not the total fixation duration, reflects dependencies on the interest assessment (H(6, 343) = 15.098, *p* = 0.020).

Thus, the structure of the relationship between the eye movement metrics and subjective assessment profoundly differs for the TEXT + PICTURE and TEXT types of stimuli. In this regard, the first fixation duration metric is a much stronger predictor than the total fixation duration metric for the text area.

The picture area total fixation duration was also analyzed for the type A to assess its dependencies on subjective rating scales for TEXT + PICTURE stimuli. Significant differences were found concerning the assessment of novelty (H(6, 326) = 17.230, *p* = 0.008) and emotions (H(6, 326) = 13.970, *p* = 0.030). No significant differences were found in the evaluations of the familiarity, complexity, cognitive, value, and interest assessments.

Correlations between the text and picture areas total fixation duration and the subjective content assessment are presented in Appendix A. No correlations (rho > 0.3) were found for both the total viewing time and text area total time, indicating a nonlinear nature of the relationships.

## 4. Discussion

Interest is a positive emotion that is important for activating adaptive behavior and maintaining mental health (Fredrickson 2004; Izard 2009; Hu et al. 2017). It is a key factor that stimulates learning motivation, ensures more effective engagement of attention and memory resources, as well as providing positive reinforcement when acquiring new information (Reeve et al. 2015; Jansen et al. 2016; Renninger and Hidi 2019, 2022). In our study, we explored the possibilities of using the eye-tracking method to detect interest in educational content, considering its multimedia nature and specificity.

The study results demonstrate several insights into the area under consideration. First of all, the materials used exhibit several characteristics compared to known literary data (Johnson and Mayer 2012; Mayer et al. 2014; Mason et al. 2017; Lindner et al. 2021). Multimedia materials attracted more visual attention, but reading time did not significantly change. Additionally, the number of correct answers did not significantly differ between the two types of materials. Similarly, no differences were found in the subjective assessment. However, even in such conditions, a clear advantage of the multimedia materials used (‘multimedia effect’) was observed compared to purely textual ones in the early stages of information processing. Multimedia stimuli were less frequently judged as completely new, and the number of correct answers was lower in such assessments compared to the textual format. The results obtained suggest that the presence of an image enhances more effective orientation in information regarding the assessment of its novelty. This contributes to more effective retrieval from long-term memory and extraction of relevant information on one hand. On the other hand, relevant information may be absent or inaccessible, which can lead to a decrease in the proportion of correct answers when the stimulus is assumed to be completely new.

Second, despite the fact that interest is more often associated with completely new information, obtained correlations suggest that interest is a result of the balance between familiarity with the topic and its novelty. It is shown that an intermediate level of text complexity may be more preferable, while texts that are either too easy or too difficult can be demotivating (Lepper et al. 2022). In this regard, it is worth noting that an intermediate level of motivation is associated with the highest effectiveness (Yerkes and Dodson 1908). Based on our research findings, the intermediate level of difficulty, however, was associated with a lower proportion of correct answers for textual materials, which is a counterintuitive result and requires further in-depth investigation.

Third, the results of this study indicate that direct correlations with various components of subjective content assessment are virtually absent. At the same time, there is a stronger coherence among the subjective scales themselves. This suggests that interest has a complex nature and remains incompletely understood. The information processes underlying interest, with their objective markers related to the distribution of visual attention, require further in-depth examination. Despite this, the obtained data align well with theoretical investigations, particularly the four-phase model (Hidi and Renninger 2006; Renninger and Hidi 2019, 2022). According to it, interest is composed of affective, antecedent knowledge, and significance components, thus representing an emotional–cognitive schema. Specific characteristics of “objects of interest” can influence their emotional evaluation: novelty, typicality, complexity, familiarity with the subject, clarity, contrast, and symmetry (Thai et al. 2019; Mungan et al. 2019). The preference for a particular stimulus, as expressed in behavior, is not described from the perspective of a single characteristic; it is assumed that a “collective measure” exists, where respondents combine different features when making affective judgments (Berlyne 1974; Mungan et al. 2019). Emotional interest or cognitive interest can be elicited, but both components are involved in forming interest (Hidi and Renninger 2006). There is also evidence indicating the possibility of stimulating interest through perceived significance (Wang et al. 2021). The closest connection of interest is with cognitive and emotional components, as observed in our study, aligning with the idea that interest can be independently triggered by these elements.

Lastly, one result found is that the first fixation duration on the text area of TEXT + PICTURE demonstrates a dependency on the subjective interest assessment in a non-linear manner. The first fixation duration tends to be lower with a higher interest assessment, except for edge ratings. This indicates a reduction in the time spent on initial information processing when reading text on the topic of interest. Presumably, the reduction in verbal information processing time is associated with a richer vocabulary on the topic of interest, due to accumulated experience. This analogy can be drawn from literature data where the literacy level and vocabulary richness are associated with shorter fixation durations. The aggregate of prior and acquired knowledge and experience is linked to the development of interest and the readiness to interact with tasks (Krapp 2002; Sui and Humphreys 2015; Fryer and Ainley 2019; Nuutila et al. 2020; Fryer et al. 2021; Lepper et al. 2022). Even the emergence of a new interest cannot be perceived as completely new personality–object relations; rather, interest is built on structural and dynamic components acquired in earlier stages of ontogenesis (Krapp 2002). Humans are characterized by attentional bias: the processing of self-related information linked to the functioning of the reward system plays a significant role in memory, perception, and decision-making (Sui and Humphreys 2015). An important result is that an individual’s history of interaction with specific topics emerges as a key factor determining the ongoing information processing reflected in eye movement metrics. In cases where interest is studied within a narrow topic, correlations between eye movement metrics and interest are found at a higher level than in this study, which reflects a more realistic scenario where topics are diverse.

It is known that prior cooking interest positively correlates with the total fixation duration and total fixation duration on the text area of a static multimedia recipe (Wang et al. 2016). Comparing these results allows us to conclude that the manifestation of interest in eye movement behavior may depend not only on the aforementioned factors but also on the task context.

Such adaptive behavioral changes driven by interest open up prospects for developing more sophisticated methods based on eye-tracking to understand the dynamics of informational and motivational processes during the interaction with educational materials. For example, neural network models that account for associated processes with interest and their diverse impacts on eye movement metrics could theoretically not only determine the presence of interest but also identify factors influencing why a particular stimulus evokes or fails to evoke interest in the context of a specific educational environment.

In summary, the results obtained demonstrate the potential role of eye-tracking as a tool for objectively evaluating educational content within the framework of learning. The perspective is more closely related to multimedia content, which more effectively activates not only the cognitive component but also the emotional component of interest, which is particularly important for interest formation. Developing solutions based on eye-tracking may contribute to integrating feedback systems into the education system in real or pseudo-real time to enhance the efficiency of the educational process.

## 5. Limitations

In the present study, physiological correlates related to content characteristics, its presentation format, and respondent interest were identified. Physiologically measurable data during content perception can serve as an objective measure of the human functional state during reading, and their potential application as a tool for assessing complex mental processes opens up avenues for further research in this field.

However, the study’s format imposes limitations on the interpretation of the obtained results and conclusions. For instance, the procedure itself was conducted within a laboratory setting with a large amount of equipment, rather than in the familiar environments of educational classrooms or workplaces for each respondent, thereby reducing the external validity of the data obtained.

Additionally, the authors have not yet developed a comprehensive interpretive model that would account for a wide range of factors, including all sociodemographic and psychological indicators of the study participants.

The repeatability of the results has not yet been verified across different age ranges and various professional profiles.

Furthermore, it would be intriguing to test the repeatability of the results on other textual and graphical materials, as well as in the context of real educational processes with new participant samples.

## Figures and Tables

**Table 1:** Means of the Likert scale rating for seven subjective assessment scales with standard deviations, and correlations with confidence intervals.

Variable	*M* (*SD*)	Q1	Q2	Q3	Q4	Q5	Q6
Q1. familiarity	4.04 (2.01)						
Q2. novelty	4.73 (2.01)	−.79 **					
		[−.81, −.76]					
Q3. complexity	3.19 (1.70)	−.53 **	.37 **				
		[−.58, −.48]	[.31, .43]				
Q4. cognitive	4.35 (1.72)	.38 **	−.19 **	−.55 **			
		[.32, .44]	[−.26, −.13]	[−.59, −.50]			
Q5. value	4.09 (1.78)	.46 **	−.34 **	−.48 **	.75 **		
		[.41, .51]	[−.40, −.28]	[−.53, −.43]	[.72, .78]		
Q6. emotions	4.42 (1.58)	.37 **	−.20 **	−.62 **	.75 **	.61 **	
		[.31, .42]	[−.27, −.14]	[−.66, −.58]	[.72, .78]	[.57, .65]	
Q7. interest	4.64 (1.64)	.31 **	−.11 **	−.69 **	.74 **	.56 **	.77 **
		[.25, .37]	[−.17, −.04]	[−.73, −.66]	[.71, .77]	[.51, .61]	[.74, .80]

** indicates *p* < .01. M and SD are used to represent mean and standard deviation, respectively. Values in square brackets indicate the 95% confidence interval for each correlation. M—mean, SD—standard deviation.

**Table 2:** Eye movement indicators when viewing different types of materials and the results of one-factor repeated measures analysis.

Parameter, ms	TEXT ^1^	TEXT + PICTURE ^1^	Predictor	*df_Num_*	*df_Den_*	*F*	*p*	η^2^_g_
Text area total fixation duration	242 (36)	240 (29)	(Intercept)	1	56	3604.60	0.000	0.98
			type	1	56	3.48	0.067	0.00
Text area first fixation duration	251 (33)	249 (33)	(Intercept)	1	56	3578.99	0.000	0.98
			type	1	56	2.98	0.090	0.00
Text area total viewing time	69,208 (26,054)	69,978 (25,973)	(Intercept)	1	56	600.62	0.000	0.91
			type	1	56	1.61	0.209	0.00
Total stimulus viewing time	69,208 (26,054)	75,222 (27,811)	(Intercept)	1	56	601.89	0.000	0.91
			type	1	56	43.20	0.000	0.02

^1^ The values represent M (SD).

**Table 3:** Correlations of proportion of correct answers with subjective assessment using Likert scale score.

Stimuli Type	Q1	Q2	Q3	Q4	Q5	Q6	Q7
TEXT	0.37	−0.24	−0.53	0.92 **	0.77 *	0.88 **	0.94 **
	[−0.53, 0.88]	[−0.84, 0.62]	[−0.92, 0.37]	[0.53, 0.99]	[0.04, 0.96]	[0.37, 0.98]	[0.64, 0.99]
TEXT × PICTURE	0.70	−0.72	−0.88 **	0.59	0.79 *	0.38	0.69
	[−0.11, 0.95]	[−0.95, 0.08]	[−0.98, −0.38]	[−0.29, 0.93]	[0.10, 0.97]	[−0.52, 0.88]	[−0.13, 0.95]

* indicates *p* < .05. ** indicates *p* < .01. Values in square brackets indicate the 95% confidence interval for each correlation.

## Data Availability

Data available on request due to restrictions.

## Appendix A

**Table A1:** Correlations of eye movement indicators with subjective assessments using Likert scale scores.

Parameter	Stimuli Type	Q1	Q2	Q3	Q4	Q5	Q6	Q7
Text area total fixation duration	TEXT	−0.08	0.10 *	0.07	−0.04	−0.07	−0.01	0.02
		[−0.18, 0.02]	[0.01, 0.20]	[−0.02, 0.17]	[−0.14, 0.06]	[−0.17, 0.02]	[−0.10, 0.09]	[−0.07, 0.12]
	TEXT + PICTURE	−0.12 *	0.09	0.08	−0.12 *	−0.14 **	−0.05	−0.06
		[−0.22, −0.03]	[−0.00, 0.19]	[−0.02, 0.17]	[−0.22, −0.03]	[−0.23, −0.04]	[−0.15, 0.04]	[−0.15, 0.04]
Text area first fixation duration	TEXT	−0.04	0.08	0.07	−0.10	−0.09	−0.01	0.00
		[−0.14, 0.07]	[−0.03, 0.18]	[−0.03, 0.18]	[−0.20, 0.01]	[−0.19, 0.02]	[−0.12, 0.10]	[−0.10, 0.11]
	TEXT + PICTURE	−0.11 *	0.08	0.12 *	−0.15 **	−0.15 **	−0.10	−0.12 *
		[−0.21, −0.01]	[−0.03, 0.18]	[0.02, 0.23]	[−0.25, −0.04]	[−0.25, −0.05]	[−0.20, 0.00]	[−0.22, −0.01]
Text area total viewing time	TEXT	−0.28 **	0.25 **	0.11 *	−0.04	−0.13 **	0.00	0.04
		[−0.36, −0.18]	[0.15, 0.34]	[0.01, 0.20]	[−0.14, 0.05]	[−0.23, −0.04]	[−0.09, 0.10]	[−0.06, 0.13]
	TEXT + PICTURE	−0.20 **	0.20 **	0.11 *	−0.08	−0.12 *	−0.03	−0.06
		[−0.29, −0.10]	[0.10, 0.29]	[0.02, 0.21]	[−0.18, 0.01]	[−0.21, −0.02]	[−0.12, 0.07]	[−0.15, 0.04]
Total stimulus viewing time	TEXT	−0.28 **	0.25 **	0.11 *	−0.04	−0.13 **	0.00	0.04
		[−0.36, −0.18]	[0.15, 0.34]	[0.01, 0.20]	[−0.14, 0.05]	[−0.23, −0.04]	[−0.09, 0.10]	[−0.06, 0.13]
	TEXT + PICTURE	−0.19 **	0.19 **	0.11 *	−0.07	−0.12 *	−0.02	−0.05
		[−0.28, −0.09]	[0.10, 0.28]	[0.02, 0.21]	[−0.17, 0.02]	[−0.21, −0.02]	[−0.12, 0.08]	[−0.15, 0.04]
Text-picture areas transitions	TEXT + PICTURE	0.04	−0.03	−0.04	0.07	0.09	0.11 *	0.06
		[−0.07, 0.14]	[−0.13, 0.08]	[−0.14, 0.06]	[−0.03, 0.18]	[−0.01, 0.19]	[0.00, 0.21]	[−0.05, 0.16]
Picture area total viewing time	TEXT + PICTURE	−0.06	0.06	0.05	0.03	−0.03	0.04	0.01
		[−0.16, 0.04]	[−0.04, 0.15]	[−0.05, 0.14]	[−0.07, 0.13]	[−0.13, 0.07]	[−0.06, 0.14]	[−0.09, 0.11]
Picture area total fixation duration	TEXT + PICTURE	−0.01	0.04	0.05	−0.03	−0.06	−0.07	−0.05
		[−0.11, 0.09]	[−0.06, 0.14]	[−0.05, 0.15]	[−0.13, 0.07]	[−0.15, 0.04]	[−0.17, 0.03]	[−0.15, 0.05]

* indicates *p* < 0.05. ** indicates *p* < 0.01. Values in square brackets indicate the 95% confidence interval for each correlation.
